# Food Legumes and Rising Temperatures: Effects, Adaptive Functional Mechanisms Specific to Reproductive Growth Stage and Strategies to Improve Heat Tolerance

**DOI:** 10.3389/fpls.2017.01658

**Published:** 2017-10-04

**Authors:** Kumari Sita, Akanksha Sehgal, Bindumadhava HanumanthaRao, Ramakrishnan M. Nair, P. V. Vara Prasad, Shiv Kumar, Pooran M. Gaur, Muhammad Farooq, Kadambot H. M. Siddique, Rajeev K. Varshney, Harsh Nayyar

**Affiliations:** ^1^Department of Botany, Panjab University, Chandigarh, India; ^2^World Vegetable Center, South Asia, Hyderabad, India; ^3^Sustainable Intensification Innovation Lab, Kansas State University, Manhattan, KS, United States; ^4^International Center for Agricultural Research in the Dry Areas, Rabat, Morocco; ^5^International Crops Research Institute for the Semi-Arid Tropics, Hyderabad, India; ^6^Department of Agronomy, University of Agriculture Faisalabad, Faisalabad, Pakistan; ^7^The UWA Institute of Agriculture, University of Western Australia, Perth, WA, Australia; ^8^Department of Crop Sciences, College of Agricultural and Marine Sciences, Sultan Qaboos University, Al-khod, Oman

**Keywords:** food legumes, high temperature stress, functional mechanisms, reproductive function, ‘Omics’ approach

## Abstract

Ambient temperatures are predicted to rise in the future owing to several reasons associated with global climate changes. These temperature increases can result in heat stress- a severe threat to crop production in most countries. Legumes are well-known for their impact on agricultural sustainability as well as their nutritional and health benefits. Heat stress imposes challenges for legume crops and has deleterious effects on the morphology, physiology, and reproductive growth of plants. High-temperature stress at the time of the reproductive stage is becoming a severe limitation for production of grain legumes as their cultivation expands to warmer environments and temperature variability increases due to climate change. The reproductive period is vital in the life cycle of all plants and is susceptible to high-temperature stress as various metabolic processes are adversely impacted during this phase, which reduces crop yield. Food legumes exposed to high-temperature stress during reproduction show flower abortion, pollen and ovule infertility, impaired fertilization, and reduced seed filling, leading to smaller seeds and poor yields. Through various breeding techniques, heat tolerance in major legumes can be enhanced to improve performance in the field. Omics approaches unravel different mechanisms underlying thermotolerance, which is imperative to understand the processes of molecular responses toward high-temperature stress.

## Introduction

**FIGURE 1:**
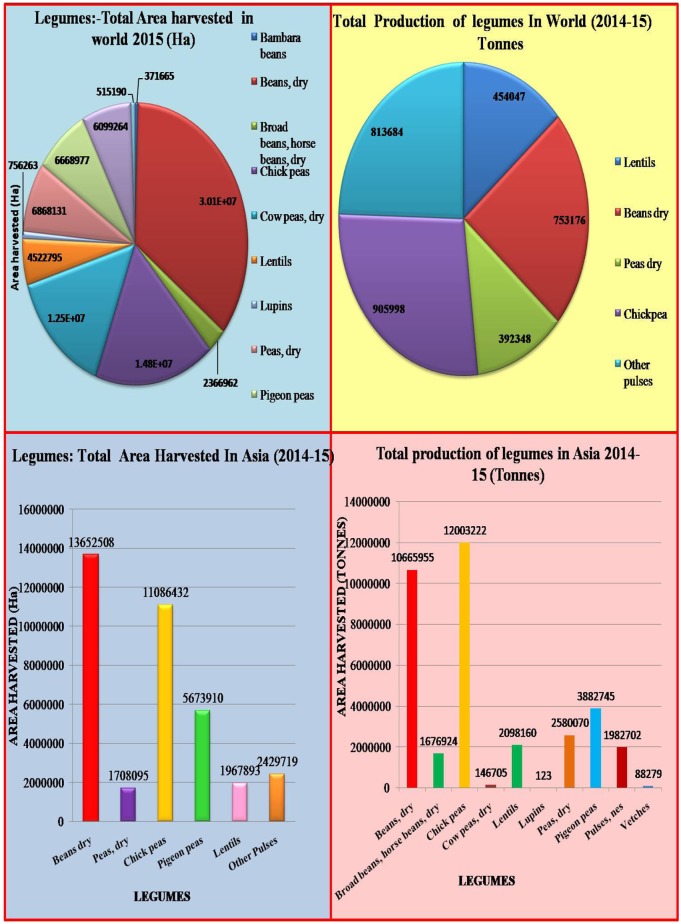
Total legume production and area harvested worldwide an in Asia in 2014–2015 (modified from FAOSTAT, 2014).

Legumes belong to the family Fabaceae/Leguminosae (with about 700 genera and 18,000 species). Legume crops can be divided into two groups according to their ability to grow in different seasons, namely cool-season food legumes and warm- or tropical-season food legumes (Miller et al., 2002; Toker and Yadav, 2010). Cool-season food legumes include broad bean (*Vicia faba*), lentil (*Lens* *culinaris*), lupin (*Lupinus* spp.), dry pea (*Pisum sativum*), chickpea (*Cicer arietinum*), grass pea (*Lathyrus sativus*), and common vetch (*Vicia sativa*) (Andrews and Hodge, 2010). Warm-season food legumes include pigeonpea (*Cajanus cajan*), cowpea (*Vigna unguiculata*), mungbean (*Vigna radiata* var. *radiata*), common bean (*Phaseolus* spp.) and urd bean (*Vigna mungo*), which are mainly grown in hot and humid conditions (Singh and Singh, 2011). Legumes rank third in world crop production, after cereals and oilseeds (Popelka et al., 2004); these crops are important source of food, feed, and fodder in several agricultural systems and are grown on a large scale in the semi-arid tropics (Popelka et al., 2004; Varshney and Dubey, 2009). The principal grain legumes, in order of their respective worldwide consumption, are common beans (*Phaseolus* spp.), field pea, chickpea, broad bean, pigeon pea, mungbean, cowpea, and lentil (Duc et al., 2015). Grain legumes alone contribute 33% of human protein nutrition and can fix atmospheric nitrogen in symbiotic association with *Rhizobium* bacteria, to fulfill the nitrogen requirement of the succeeding crop. Legumes are cultivated in crop rotation worldwide along with other crops but their production potential is constrained by high temperatures (McDonald and Paulsen, 1997; Considine et al., 2017). Legume production and harvested area worldwide and in Asia in 2014–2015 are shown in **Figure 1**.

Various abiotic stresses, such as temperature, drought and salt, affect the growth of legumes at different developmental stages (Suzuki et al., 2014). Abiotic stresses are the primary cause of crop losses worldwide, reducing the yield of most plants by >50% (Rodríguez et al., 2006). Abiotic stresses result in a series of morphological, physiological, biochemical and molecular alterations, which negatively influence plant growth, productivity and yield (Wang et al., 2001; Bita and Gerats, 2013). Plants experience multiple effects of these stresses including physiological functions such as photosynthesis, respiration, nitrogen fixation, reproduction, and oxidative metabolism (Iba, 2002; Farooq et al., 2008). Temperature stress has the widest and most far-reaching effects on various crops leading to a severe reduction in yield potential (Bita and Gerats, 2013). This review emphasizes responses and adaptations of various food legumes to heat stress—focusing on the reproductive phase—intrinsic tolerance mechanisms and strategies toward the genetic improvement of legume crops to heat stress.

## High-Temperature Stress and Its Threshold in Plants

Temperature is a major factor affecting seed yield and quality in legumes (Ruelland and Zachowski, 2010; Christophe et al., 2011). Increases in air temperature, even by one degree above a threshold level, is considered heat stress in plants (Teixeira et al., 2013). Heat stress for most subtropical and tropical crops is when temperatures increase above 32–35°C (Bita and Gerats, 2013); however, a daily maximum temperature above 25°C is considered the upper threshold for heat stress in cool-season crops (Wahid et al., 2007). The impact of heat stress depends on the intensity, duration of exposure, and the degree of the elevated temperature. Extreme variations in temperature, both high and low, can have serious implications on plant development by impairing plant growth and function (Wahid et al., 2007). Temperature stress imposes challenges in plants at various organizational levels with deleterious effects on vegetative and reproductive growth (Hamidou et al., 2013). Furthermore, increased frequency of temperature stress can disrupt the physiological processes of plants resulting in photosynthetic inhibition, reduced nitrogen anabolism, higher protein catabolism, and accumulation of the end products of lipid peroxidation (Jagtap et al., 1998; Jiang and Huang, 2001a,b). Heat-stressed plants show shorter vegetative and pod-filling periods (Adams et al., 2001), poor crop stand and consequently reduced yield. High-temperature stress affects reproductive development, as reported in legumes such as chickpea (Kaushal et al., 2013; Kumar et al., 2013), pea (Guilioni et al., 1997), common bean (Gross and Kigel, 1994; Vara Prasad et al., 2002), mungbean (Tzudir et al., 2014; Bindumadhava et al., 2016), cowpea (Ahmed et al., 1992) and cereals such as rice (*Oryza sativa*; Madan et al., 2012), wheat (*Triticum aestivum*; Wahid et al., 2007), barley (*Hordeum vulgare;* Barnabás et al., 2008), and maize (*Zea mays*; Kumar et al., 2012a). High temperature negatively affects flower initiation, pollen viability (germination and tube growth), stigma receptivity, ovule viability, ovule size, fertilization, seed/fruit set, seed composition, grain filling, and seed quality (Barnabás et al., 2008). Cool-season food legumes are more sensitive to heat stress than warm-season food legumes. The critical temperature for heat tolerance seems to be higher in chickpea than in faba bean, lentil, and field pea, and the reverse is true for cold tolerance (Devasirvatham et al., 2013). The threshold temperatures of various legume crops are shown in **Table 1**.

**Table 1:** The heat stress threshold temperature range of some leguminous crops.

Legume crop	Threshold temperature (°C)	Reference
Chickpea	15–30	Kaushal et al., 2011
Common bean	20–24	Kigel et al., 1991; Vara Prasad et al., 2002
Cowpea	18–28	Laing et al., 1984; Craufurd et al., 1997
Faba bean	25	Bishop et al., 2016
Groundnut	30–35	Vara Prasad et al., 2001; Hatfield et al., 2008
Lentil	15–30	Barghi et al., 2012
Lupins	20–30	Bordeleau and Prévost, 1994
Mungbean	28–35	Kumar et al., 2011
Pea	15–25	Gladish and Rost, 1993
Pigeon pea	18–30	Duke, 1981; FAOSTAT, 2014
Soybean	23–26	Boote et al., 1998
Urd bean	25–35	Shirsath and Bhosale Agro India Ltd, 2017


## Heat Stress Sensing and Signal Transduction

Plants detect even mild increases in temperature due to presence of sensing mechanisms on their membranes (Wise et al., 2004). Under high-temperature stress, membranes show increase in fluidity, which is detetced by membrane sensors resulting in conformational changes and phosphorylation/dephosphorylation events (Kaushal et al., 2016; Sehgal et al., 2016). Four sensors are reported to perceive heat stimulus (Mittler et al., 2012), which include plasma-membrane-bound Ca^2+^ channels (Saidi et al., 2009), two unfolded protein sensors—one in the endoplasmic reticulum (ER) (Deng et al., 2011; Srivastava et al., 2014) and the other in the cytosol (Sugio et al., 2009), and a histone sensor in the nucleus (Kumar and Wigge, 2010).

**FIGURE 2:**
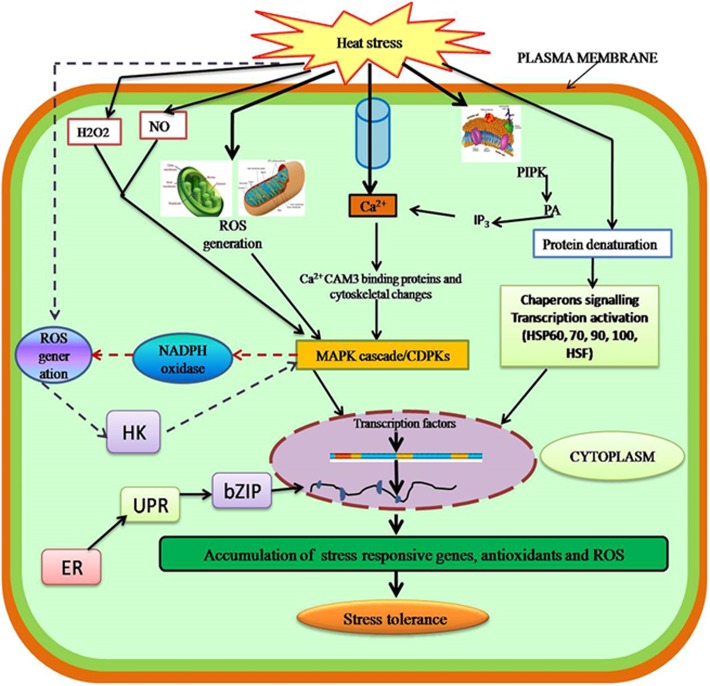
Sensing and signaling in plants in response to heat stress. Heat stress affects the plasma membrane to activate calcium channels, which induces Ca^2+^ influx and activates the heat shock response. Thus, the MAPK cascade leads to gene expression. Secondary signals such as ROS, H_2_O_2_, NO, and ABA lead to stress tolerance. CaM3, calmodulin; HSFs, heat shock factors; CDPKs, calcium-dependent protein kinases; MAPKs, mitogen-activated protein kinases; ROS, reactive oxygen species; NO, nitric oxide; HK, histidine kinase; UPR, unfolded protein response; ER-UPR, endoplasmic reticulum unfolded proteins; Cyt-UPR, cytosolic unfolded proteins.

Most studies have revealed that moderate increases in temperature are initially sensed by plasma membrane leading to the activation of Ca^2+^ channels, which causes an inward flux of Ca^2+^ into cells to activate the heat shock response (HSR) (Bokszczanin and Fragkostefanakis, 2013). The inward flux of Ca^2+^ is an important indicator of heat stress as indicated by various pathways including calcium channel blockers or chelators. In plants, this inward flux of Ca^2+^ regulates various signaling pathways. AtCaM3 (a calmodulin) is required for heat stress signaling as reported in *Arabidopsis thaliana* (Liu et al., 2008; Zhang et al., 2009), which in turn activates the various transcriptional factors such as WRKY39 (Li et al., 2010) and heat shock transcription factors (HSFs) (Liu et al., 2011). Moreover, Ca^2+^ influx leads to the activation of several calcium-dependent protein kinases (CDPKs), which in turn activate various mitogen-activated protein kinases (MAPKs) (Sangwan et al., 2002) or the reactive oxygen species (ROS)-producing enzyme NADPH oxidase (**Figure 2**) (Suzuki et al., 2011). The Ca^2+^/calmodulin binding protein kinase (CBK) is also activated by AtCaM3, which phosphorylates members of the HSF family such as HSF1a (Liu et al., 2008). Heat stress activates lipid signaling where phospholipase-D (PLD), phosphatidylinositol-4-phosphate-5-kinase (PIPK), and various other lipid signaling molecules such as phosphatidic acid, phosphatidylinositol-4,5-bisphosphate (PIP2), and D-myo-inositol-1,4,5-triphosphate (IP3) (Mishkind et al., 2009) are activated.

Heat stress also activates unfolded protein response (UPR) signaling pathways in cells. Two UPR pathways operate in plant cells, one in the ER and the other in the cytosol (Sugio et al., 2009; Pincus et al., 2010; Deng et al., 2011).

Activation of the ER UPR pathway leads to proteolytic cleavage and the release of different bZIP transcription factors (Tfr) from the ER membrane (Che et al., 2010; Deng et al., 2011). These transcription factors enter the nucleus and activate the transcription of specific genes, which in turn leads to the accumulation of ER chaperone transcripts and activation of brassinosteroid signaling (Che et al., 2010). Unfolded proteins in the cytosol trigger the cytosolic UPR pathway, which is regulated by HSF, HSFA2, and bind to HSF-binding elements in the promoters of HSR genes (Sugio et al., 2009).

High-temperature stress leads to histone acetylation, methylation, phosphorylation, ubiquitination, glycosylation, ADP-ribosylation, and sumoylation (Clapier and Cairns, 2009). The active or repressed state of the associated DNA sequence is regulated in a code-like manner by the above-listed modifications of amino-terminal histone tails protruding from the nucleosome (Jenuwein and Allis, 2001; Li et al., 2010).

## Vegetative Stage

Heat stress primarily influences the rate of plant development, which increases to a certain point and diminishes afterward (Howarth, 2005; Wahid et al., 2007). Seed germination is fundamentally reliant on temperature (Hasanuzzaman et al., 2013). Declined germination percentage, seedling emergence, abnormal seedlings, poor seedling vigor, and reduced radical and plumule growth in germinated seedlings are major impacts of heat stress in various legume crops (Hasanuzzaman et al., 2013). The temperature that seeds germinate best depends largely on plant species; for example, soybean performs best at 10–35°C, maize at 10–40°C, and wheat at 20–40°C (Probert, 2000). Reduced seed germination at high temperatures has been reported in many legumes including soybean (Ortiz and Cardemil, 2001; Ren et al., 2009), pea (Nemeskeri, 2004; Ren et al., 2009), lentil (Chakraborty and Pradhan, 2011), mungbean (Kumar et al., 2011; Devasirvatham et al., 2012a), and chickpea (Kaushal et al., 2011; Piramila et al., 2012). A study by Nemeskeri (2004) on heat tolerance in three prominent legumes (beans, pea, and soybean) revealed that exposure to 28°C for 8 days seedling stage resulted in 50.4 and 36.2% dead seeds in non-irrigated soybean and beans, respectively, and 87.6 and 36.8% in irrigated soybeans and beans, respectively. Similarly, seed germination and vigor index in mungbean seeds exposed to 10, 20, and 30 min of 50°C decreased significantly (Piramila et al., 2012). In lentil, seeds exposed to 35–40°C for 4 h had reduced germination and retarded seedling growth (Chakraborty and Pradhan, 2011).

Vegetative plant parts show various morphological symptoms in response to heat stress, such as scorching and sunburning of leaves, twigs, branches and stems, senescence of leaves followed by abscission, inhbition of shoot and root growth, and discoloration of fruits, which can severely reduce yield (Bita and Gerats, 2013). Heat stress also causes leaf wilting, leaf curling, leaf yellowing, and reduced plant height and biomass (Siddiqui et al., 2015). Exposure of plants to severe high temperature often reduces shoot growth, root growth, root number, and root diameter (Xu et al., 2000). Heat stress severely affects vegetative growth in legumes such as peanut (29 and 33°C) (Bolhuis and De Groot, 1959), pea (28–30°C) (Poehlman, 1991), and chickpea (22–25°C) (Singh and Dhaliwal, 1972). Heat stress results in water loss from cells, reduced cell size and growth, and hence reduced leaf area and biomass. When growing conditions are favorable, plants continue vegetative growth without setting pods or filling fewer pods (Davies et al., 1999; Liu et al., 2003). High temperature can severely reduce the length of the first internode resulting in premature death (Reddy et al., 2003).

## Reproductive Stage

High temperature stress affects reproductive development in legumes such as chickpea (Kaushal et al., 2013; Kumar et al., 2013), mungbean (Tzudir et al., 2014; Kaur et al., 2015), and lentil (Bhandari et al., 2016; Sita et al., 2017). The reproductive phase is divided into flower initiation, differentiation of male and female floral parts, micro and megasporogenesis, development of male and female gametophytes (pollen grain and embryo sac), pollination, micro and megagametogenesis, fertilization and seed development. Each stage responds differently to high-temperature stress, but collectively all responses result in undesirable effects and reduce net yield (Thakur et al., 2010). The phenology of a crop differs with species, sowing season, particular area, and atmospheric phenomenon (Anbessa et al., 2006). Most yield losses are related to metabolic alterations due to heat stress, reduction of developmental stages in terms of time and size, and the consequent reduction in light interception over the shortened life cycle. The processes related to carbon assimilation (photosynthesis and respiration) are also disrupted markedly, which may result in deformed and smaller organelles (Maestri et al., 2002; Barnabás et al., 2008).

**FIGURE 3:**
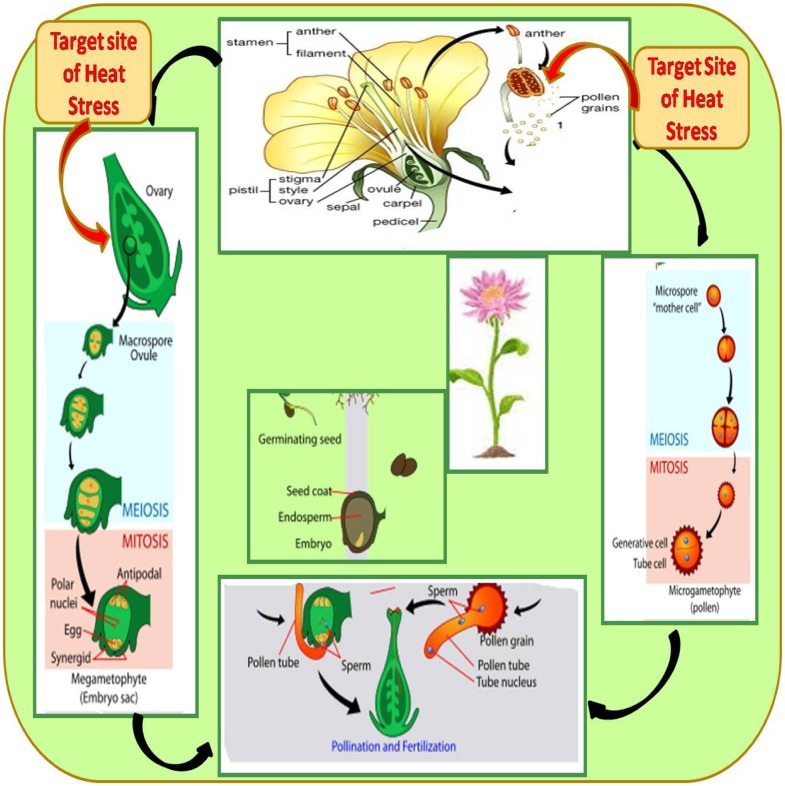
The life cycle of a typical angiosperm showing target sites of heat stress. The sporophyte phase is the main phase, which generates microspores that produce pollen grains as the male gametophytes (microgametophyte), and megagametophytes (megaspores), which form an ovule that contains female gametophytes.

Reproductive growth is more sensitive and causes various effects such as depletion of buds, flowers, fruits, pods, and seeds to result in marked reductions in yield potential (Thakur et al., 2010; Kaushal et al., 2016). Heat stress influences crop yield by impacting reproductive components during development that contribute to a reduction in harvest index and these responses differ with the severity and duration of the stress (Hedhly et al., 2009; Harsant et al., 2013). Heat stress reduces the number of flowering branches and thus the number of flowers per plant (Vara Prasad et al., 2001, 2002; Young et al., 2004; Harsant et al., 2013). Heat stress disrupts male and female gametophytes, results in poor pollen viability, poor pollen germination, inhibition of pollen tube growth, loss of stigma receptivity and ovule function, fertilization arrest, limited embryogenesis, decreased ovule viability, increased ovule abortion and poor seed set (Kumar et al., 2013; Gupta et al., 2015) (**Figure 3**).

### Flowering Initiation and Development

**FIGURE 4:**
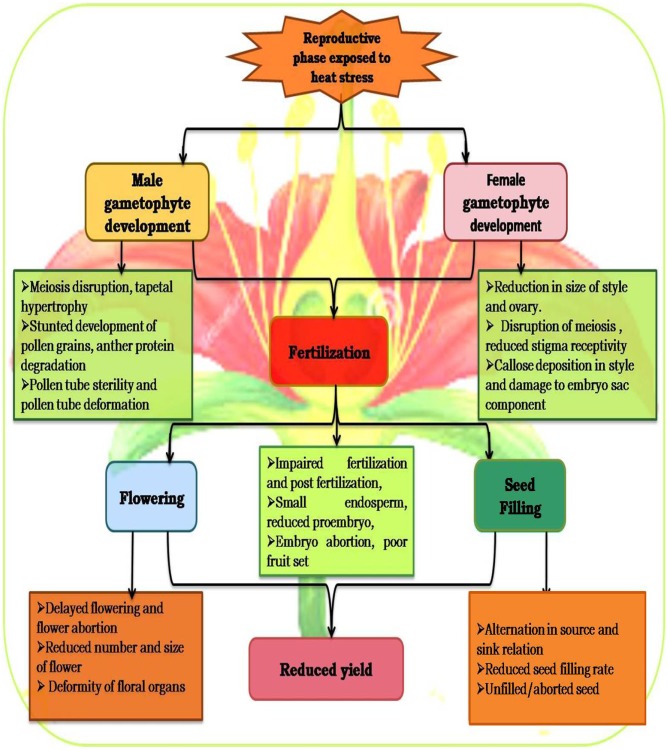
Effects of heat stress during the reproductive phase (at different functional stages).

During flower development, male and female organs are sensitive to high temperature, especially ≥30°C (**Figure 4**; Lavania et al., 2015). Heat stress severely affects flower bud initiation, and this sensitivity prevails for 10–15 days (Hedhly et al., 2009; Bita and Gerats, 2013) as reported in faba bean (Bishop et al., 2016), common bean (Vara Prasad et al., 2002), and soybean (Kitano et al., 2006). Heat stress influences the reproductive stage by decreasing the number and size of flowers, deforming floral organs, resulting in loss of flowers and young pods, and hence reduction in seed yield (Morrison and Stewart, 2002), as reported in chickpea and mungbean (Tickoo et al., 1996), common bean (Gross and Kigel, 1994; Suzuki et al., 2001), cowpea (Hall, 1992), pea (Stanfield et al., 1966), and peanut (Vara Prasad et al., 1999a). A mild heat stress during floral development severely reduced yield in faba bean (Bishop et al., 2016). The flowering stages are more susceptible to heat stress, and high temperatures are likely to coincide with gametophyte development and anther dehiscence in faba bean and some other legume species (Bishop et al., 2016).

### Meiosis and Gametophyte Development

Meiosis is an important stage in the sexual life cycle of a plant to allow the diploid sporophytic cells to produce haploid gametophytes (Thakur et al., 2010). After the inception of meiosis, the sensitivity of the male gametophyte to stress increases dramatically, with negative consequences for anthesis, pollen fertility, pollination, female fertility, early zygote development, and seed yield (Boyer and McLaughlin, 2007). In microsporogenesis of chickpea, meiosis and pollen development are most affected by heat stress (Devasirvatham et al., 2012a). Sexual reproduction and flowering, in particular, are extremely sensitive to heat stress, and often results in reduced crop productivity (Thakur et al., 2010; Bita and Gerats, 2013). Heat stress mainly accelerates the onset of anthesis, thereby initiating the reproductive stage prior to the accumulation of sufficient resources (Zinn et al., 2010; Bita and Gerats, 2013).

### Male Gametophyte

**FIGURE 5:**
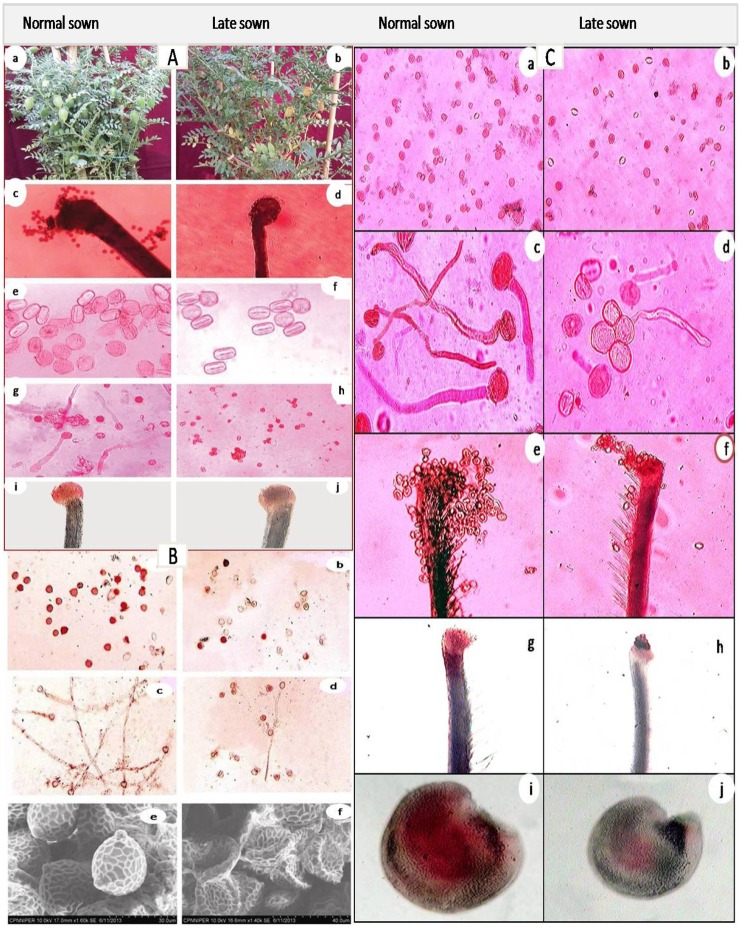
Effect of heat stress in normal-sown and late-sown (heat-stressed) plants Chickpea [(**A**: Biomass in control (a) and heat-stressed (b), Pollen load in control (c) and heat-stressed (d), Pollen viability in control (e) and heat-stressed (f) pollen viability in control (g) and heat-stressed (h), Stigm receptivity in control (i) and heat-stressed (j) (Kaushal et al., 2013)], Mungbean [(**B**; Pollen viability in control (a) and heat-stressed (b), pollen germination in control (c) and heat-stressed (d), and SEM observations on pollen morphology in control (e) and heat-stressed (f) (Kaur et al., 2015)], and lentil [(**C**; Pollen viability in control (a) and heat-stressed (b), Pollen germination in control (c) and heat-stressed (d), Pollen load in control (e) and heat-stressed (f), stigma receptivity in control (g) and heat-stressed (h), ovule viablity in control (i) and heat-stressed (j)]. Notice reduction in pollen load, pollen viability, *in vitro* pollen germination, stigma receptivity and ovule viabilty in heat-stressed plants of all the legumes (Kaushal et al., 2013; Kaur et al., 2015). Figures are being reproduced with the permission from the copyright holder.

Male reproductive development in higher plants is very sensitive to heat stress at all growth stages (Bita and Gerats, 2013; Sage et al., 2015). In particular, high temperature stress results in a lower seed set due to male sterility in most legume crops, including chickpea (Devasirvatham et al., 2012a), common bean (Monterroso and Wien, 1990), cowpea (Warrag and Hall, 1983), and field pea (Jiang et al., 2015). In most legumes, the male gametophyte is more sensitive to high temperature than the female gametophyte (Devasirvatham et al., 2012a; Sage et al., 2015; Bhandari et al., 2016). Development of the male gametophyte (pollen grains) starts with the separation of reproductive tissue from the anther, followed by meiosis of the pollen mother cell, mitosis and microspore maturation, and the formation of mature pollen grains (Bita and Gerats, 2013). Specialized anther tissue has non-reproductive (tapetum for support, stomium for dehiscence) or reproductive functions (pollen mother cell for pollen formation). Male fertility depends on both the status of the tapetum and microspore development (Zinn et al., 2010; Bita and Gerats, 2013). Heat stress alters gene expression, which is possibly connected to tapetum degeneration and pollen sterility, in most plant species (Oshino et al., 2007; Endo et al., 2009). Sakata et al. (2010) suggested that understanding heat stress effects on pollen development will involve observations on carbohydrate turnover during this stage. Mature pollen grains are more tolerant to heat stress than any other stage of male gametophyte development (Hedhly, 2011). Tolerance of pollen grains to high temperature may be associated with its low plasma content, low metabolic activity to its protective structures, or its carbohydrate content and dynamics (Kaushal et al., 2013; **Figure 5**). Pollen grains penetrate the stigmatic surface, and pollen tube growth starts within the style and within the ovary toward the female gametophyte; the pollen tube growth rate is the first and most important characteristic to check under heat stress (Hedhly, 2011). Heat stress affects male sterility in most sensitive crop plants, by impairing pollen development to severely reduce yield (Wassmann et al., 2009; Bita and Gerats, 2013), as reported in cowpea (Ahmed et al., 1992), chickpea (Devasirvatham et al., 2012a, 2013; Kaushal et al., 2013), common bean (Gross and Kigel, 1994), groundnut (Vara Prasad et al., 1999b), soybean (Djanaguiraman et al., 2013), chickpea (Devasirvatham et al., 2013), field pea (Jiang et al., 2015), and faba bean (Bishop et al., 2016). Developing anthers are a strong resource sink and heat stress affects the development of tapetum cells and microspores, which involve DNA, carbohydrates, proteins, and lipids synthesis (Ma, 2005; Sage et al., 2015). Tapetal cells and microspores are separated symplastically from other anther tissue, and tapetal cells are metabolically highly active to nourish the growing microspores. The high transport and metabolic activity of the tapetum layer is indicated by the presence of some cell organelles such as plastids, mitochondria, peroxisomes, and endomembrane and cytoskeleton systems involved in processing and transporting metabolites (Bagha, 2014). Suzuki et al. (2001) found that heat stress caused early degeneration of the tapetum layer and disrupted ER in *Phaseolus vulgaris*.

Heat stress delinks source and sink strength leading to depletion of available carbohydrates at the reproductive stage of plant development, ultimately reducing fruit set and other yield attributes in chickpea (Nayyar et al., 2005; Kaushal et al., 2013) (**Figure 5**) and lentil (Bhandari et al., 2016; Sita et al., 2017). High temperature also influences early abortion of tapetal cells which leads to pollen sterility (Parish et al., 2012), structural abnormalities in developing microspore-associated tapetal degeneration due to deformity in ER (Peet et al., 2002), fertilization arrest and abrupt embryo development (Barnabás et al., 2008), reduced seed germination, loss of vigor, and reduced seedling emergence in many crop plants (Akman, 2009; Ren et al., 2009; Bita and Gerats, 2013). Heat stress results in premature abortion of tapetal cells causing the pollen mother cells to rapidlly progress toward meiotic prophase and undergo programmed cell death (PCD) resulting in pollen sterility (Sakata and Higashitani, 2008; Parish et al., 2012). For example, the structural abnormalities in developing microspores of snap bean anthers under heat stress were associated with degenration of tapetum as a result of malformations in the ER (Suzuki et al., 2001). Heat stress caused reduction in dehiscence of anthers, accompanied by closure of the locules, and thus decrease in pollen dispersal in several crop plants (Peet et al., 2002). Exposure to high temperature after fertilization can impair subsequent embryo development (Barnabás et al., 2008). The reproductive failures in chickpea due to high-temperature stress are the result of disrupted sucrose metabolism in leaves as well as anthers (Kaushal et al., 2013).

### Female Gametophyte

The female gametophyte in plants is also called the embryo sac and is mostly a seven-celled structure (Thakur et al., 2010). Female gametophyte development occurs over two stages referred to as megasporogenesis and megagametogenesis. The female gametophyte is less sensitive to heat stress than the male gametophytic (Kaushal et al., 2013, 2016). Elevated temperatures probably inhibit style length and consequently induce abnormalities in ovary development, as observed in chickpea (Srinivasan et al., 1999). Temperatures >30°C reduce stigmatic receptivity and stigmatic pollen germination (Harsant et al., 2013), stigma and style growth (Snider et al., 2011; Song et al., 2015), and ovule penetration (Saini et al., 1983). Heat stress abruptly affects almost all aspects of female gametophyte development, e.g., reduced stigma receptivity in chickpea at 40/30 and 45/35°C (Kumar et al., 2013), and reduced ovule number and viability in common bean at 30°C (Suzuki et al., 2001). The female gametophyte produces important cells within the ovule *viz*. egg, central cell and synergids, which are developed by mitotic divisions (Sage et al., 2015). Synergids produce attractants into the micropylar end that guide pollen tube growth to the ovule (Chae and Lord, 2011). Heat stress alters the secretion of pollen tube attractants (Higashiyama et al., 1998), and reduces penetration of the ovule by the pollen tube (Saini et al., 1983). The effects of heat stress on expansion, division, and differentiation of egg and synergids in female gametophytes have been reported in bean (Sage et al., 2015).

Both male and female plant parts coordinate to make certain the deposition of pollen when the stigma becomes receptive, and this involves appropriate positionining of anthers nearby to the stigma for capturing the pollen after dehiscence (Sage et al., 2015). Heat stress disrupts this coordination by changing the structural positioning of anthers related to the stigma, the timing of dehiscence of anthers, and maturation and recetivity of stigma/style due to alteration in cell division and elongation (Basra, 2000; Giorno et al., 2013; Sage et al., 2015). These changes ultimately prevent pollen deposition on the stigma and alter the fertilization process.

### Pollination and Fertilization

For establishment of seed, the pollen grains must interact with a receptive stigma, followed by pollen tube growth to reach the ovules for fertilization, and embryo and endosperm development (Barnabás et al., 2008). Some of these events may be impacted by the adverse environmental conditions frequently encountered by crop plants (Driedonks et al., 2016). High temperature may arrest fertilization by inhibiting the development of male (Jain et al., 2007) and female gametophytes (Snider et al., 2009). Reduced fertilization is a common problem associated with heat due to disruption of meiosis and fertilization in various species, such as chickpea, cowpea, and barley (Kaushal et al., 2013; Jagadish et al., 2014; Bac-Molenaar et al., 2015; Driedonks et al., 2016). Reduced fertilization efficiency due to heat stress has been attributed to increasing oxidative stress, reduced carbohydrates, ATP concentration in gynoecium and decreased leaf photosynthesis, in mungbean (Suzuki et al., 2001), soybean (Board and Kahlon, 2011), and chickpea (Kumar et al., 2013). High temperatures during pollen development limit fertilization and seed development (Porch and Jahn, 2001) by reducing the number of mature pollen grains available for pollination (Erickson and Markhart, 2002; Sato et al., 2002), which causes abnormal pollen development, and reduces the viability and germinability of available pollen grains (Firon et al., 2006; Sato et al., 2006; Jain et al., 2007).

Heat stress (>30°C) from early meiosis to pollen maturity reduces the viability of pollen grains in chickpea resulting in fertilization failure leading to reduced seed set (Saini and Aspinall, 1981; Kaushal et al., 2016). Heat stress results in abnormal anther morphology and limits anther dehiscence at anthesis (Dupuis and Dumas, 1990), and prevents the accumulation of carbohydrates in developing anthers and pollen grains, which accounts for poor pollen viability at anthesis (Porch and Jahn, 2001; Kaushal et al., 2013). Gross and Kigel (1994) reported that high temperatures of 27/32°C at sporogenesis reduced pollen viability and yield in heat-sensitive genotypes of bean, due to failed anther dehiscence, pollen sterility, low pod and seed set,. In soybean, high temperatures of 38/28°C during flowering reduced *in vitro* pollen germination. Pollen grains were deformed, with a thicker exine and a disintegrated tapetum layer (Djanaguiraman et al., 2013). In chickpea, heat stress late in the season produced more structural abnormalities in anthers and pollen grains such as changes in anther locule number, anther epidermis wall thickening and pollen sterility in sensitive genotypes ICC-4567, ICC-10685 (Devasirvatham et al., 2013). Temperatures of 35/20 and 40/25°C pre- and post-anthesis reduced pollen viability, pollen production per flower and percentage of pollen germination in chickpea (Devasirvatham et al., 2012b). The effects of heat stress on both male and female reproductive tissue in some legume crops are shown in **Table 2**.

**Table 2:** Effect of heat stress on both reproductive function, male and female reproductive tissue in some legume crops.

Crop species	Temperature stress	Effects	Reference
Soybean	Above 35°C	Flower abscission, reduced yield	Koti et al., 2005; Salem et al., 2007
Soybean	26°C	Reproductive development	Boote et al., 1998
	23°C	Post-anthesis	Boote et al., 2005
	30.2°C	Pollen germination	Boote et al., 2005
	36.1°C	Pollen tube growth	Hatfield et al., 2008
Cowpea	33/30°C	Anther indehiscence due to degeneration of tapetal cells	Ahmed et al., 1992
Common bean	33/29°C	Degeneration of tapetal cells	Suzuki et al., 2001
Soybean	38/28°C	Decreased *in vitro* pollen germination	Djanaguiraman et al., 2013
Common bean	33/30°C	Anther indehiscence due to degeneration of tapetal cells	Ahmed et al., 1992
Chickpea	33/27°C	Anther indehiscence and pollen sterility	Gross and Kigel, 1994
Chickpea	35/20°C	Lack of pollen germination and tube growth in style	Devasirvatham et al., 2010
Chickpea	32/26°C	Abnormal embryo sac development	Polowick and Sawhney, 1987, 1988
Chickpea	45/35°C	Reduced stigma receptivity	Kumar et al., 2013
Chickpea	(≥40/30°C)	Reproductive failure, reduced yield	Gaur et al., 2014
Peanut/groundnut	29–33°C	Anthesis	Bolhuis and De Groot, 1959
		Pod, seed yield	Hatfield et al., 2008


### Seed Filling and Yield

Temperature fluctuations during seed filling drastically reduce yield in legumes such as common bean (Rainey and Griffiths, 2005), pea (McDonald and Paulsen, 1997), chickpea (Kaushal et al., 2013; Kumar et al., 2013), mungbean (Kaur et al., 2015), lentil (Barghi et al., 2012; Bhandari et al., 2016; Sita et al., 2017), and cowpea (Ahmed et al., 1992). Seed filling is the completion of growth and development in crop plants, which involves transport processes to import constituents and biochemical processes related to the synthesis of carbohydrates, proteins, and lipids in seeds (Yang and Zhang, 2006; Awasthi et al., 2014). High-temperature stress causes yield loss in legumes (Canci and Toker, 2009; Kumar et al., 2016) and other crops due to poor seed development (Hall, 2004). Moreover, heat sensitivity differs for different crop species (Sung et al., 2003); on average, a one-degree rise in temperature will reduce plant yield by at least 10%. Under high temepratures, seed filling is accelerated, to reduce the duration of this stage to limit the yield potential (Boote et al., 2005). The reduction in starch accumulation was suggested to be the primary reason of yield reduction since starch acumulation accounts for substantial dry weight of the seeds. The reduction in seed weight in response to heat stress during the early stages of seed filling can be attributed to fewer endosperm cells (Nicolas et al., 1985), while during the later stages, heat stress impairs starch synthesis by limiting the supply of assimilates to the seed (Blum, 1998) or directly affecting the synthetic processes in the seed (Yang et al., 2004).

The number of endosperm cells determined early in grain fill, and the final size of the cells influence the extent of starch and protein accumulation in each seed, the rate and duration of grain fill also affect the accumlation of the seed reserves (Egli, 1998; Barnabás et al., 2008).

Reductions in various yield attributes due to heat stress has been reported in many crops such as cowpea (Hall, 1992), pea (Guilioni et al., 1997), common bean (Vara Prasad et al., 2002; Rainey and Griffiths, 2005), peanut (Vara Prasad et al., 1999a, 2000), soybean (Board and Kahlon, 2011), lentil (Barghi et al., 2012), and chickpea (Krishnamurthy et al., 2011; Kaushal et al., 2013; Kumar et al., 2013).

High-temperature stress reduces seed size due to the insufficient accumulation of photosynthates during seed filling (Kumar et al., 2016). A few days of heat stress (30–35°C) during seed filling accelerates senescence, decreases seed set and seed weight, and reduces yield in legumes (Siddique et al., 1999; Kumar et al., 2016). High yield losses have been reported in snap bean under heat stress (Tsukaguchi et al., 2003). Gutiérrez-Rodríguez et al. (2003) studied the biomass and yield of bean plants raised in two different seasons, i.e., winter and summer, and found that the winter-sown crop had 41 and 38% higher biomass and yield, respectively than the summer-sown crop. In soybean, even short-term exposure to stressful temperatures above 40°C reduced seed production and yield (Kitano et al., 2006; Board and Kahlon, 2011; Djanaguiraman et al., 2011). Vara Prasad et al. (2006) reported that increasing temperatures from 32/22°C to 36/26°C and 40/30°C, reduced seed yield in sorghum (*Sorghum bicolor*) by 10 and 99%, respectively. High-temperature stress increased the percentage of shriveled seed and reduced seed size in common bean (Vara Prasad et al., 2002) and groundnut (Prasad et al., 2003). In chickpea, Jumrani and Bhatia (2014) reported that increased temperatures during the reproductive stage severely reduced yield (by 10, 23, 64, and 78%) at different temperature ranges (26/16, 30/18, 34/20, and 38/28°C), respectively. Kaushal et al. (2013) observed a 63–64% yield reduction in chickpea exposed to 32/20°C. In similar studies, chickpea yields declined by 34–50% (Gan et al., 2004) and 34% (Wang et al., 2006) when exposed to temperatures >32/20°C. Other studies have reported inhibitory effects of high temperature on yield in pea (McDonald and Paulsen, 1997), cowpea (Ismail and Hall, 1999; Thiaw and Hall, 2004), peanut (Prasad et al., 2003), soybean (Puteh et al., 2013), field pea (Vijaylaxmi, 2013), faba bean (Kirra et al., 2014), mungbean (Kaur et al., 2015), and lentil (Bhandari et al., 2016; Sita et al., 2017).

### Regulation of Seed Filling and Maturation

During seed filling, carbohydrates, proteins, and lipids accumulate in developing seeds (Thakur et al., 2010). Heat stress alters the activities of carbon metabolism enzymes, starch accumulation, and sucrose synthesis by down-regulating specific genes in carbohydrate metabolism (Ruan et al., 2010). Plant hormones such as ABA and cytokinins play an important role in the regulation of seed filling (Brenner and Cheikh, 1995). These phytohormones are involved in the determination of sink size and strength, and the capacity of the seed to accumulate biomass (Thakur et al., 2010). Auxins, gibberellins, and ABA mediate cell division, enlarge endosperm cells, and regulate the direction and rate of assimilate flow from source to sink tissues (Hansen and Grossmann, 2000). Heat stress can influence seed filling by changing the concentration and amount of phytohormones as well as the expression of enzymes (Thakur et al., 2010). Low leaf photosynthetic rates during seed filling in heat-stressed plants are a major cause of reduced seed size (Singh, 1987; Leport et al., 1998). The accumulation of various seed components (mainly starch and proteins) may be inhibited by heat stress due to the inactivation of enzymatic processes involving starch (Ahmadi and Baker, 2001) and protein synthesis (Triboï et al., 2003).

Auxins regulate reproductive processes; in plants, a naturally occurring auxin is indole-3-acetic acid (IAA) (Ozga et al., 2017). However, in legume species, particularly those in the Fabaceae family such as pea, grass pea (*Lathyrus sativus* L.), lentil and faba bean, also contain the naturally occurring chlorinated form of auxin, 4-chloroindole-3-acetic acid (4-Cl-IAA), which is biologically more active than IAA in auxin bioassays (Reinecke, 1999; Ozga et al., 2017). Heat stress suppresses auxin biosynthesis and signaling in developing anthers, resulting in pollen abnormalities (Higashitani, 2013; Ozga et al., 2017). Similarly, gibberellins play an important role in stamen and pollen development (Plackett et al., 2012). Some studies have revealed that jasmonic acid signaling is required for stamen development and fertility because stamen development can be restored only in jasmonic acid biosynthesis mutants by exogenous jasmonic acid (Yan et al., 2007). Elevated temperature stress affects ethylene biosynthesis/signaling pathways in developing anthers, which leads to reduced anther dehiscence. Pollen development and pollen germination can be enhanced by pre-treatment with an ethylene-releasing agent, ethephon (Firon et al., 2012).

At the stage of fruit set, high temperature reduces auxin flux through the pedicel, allowing ethylene-facilitated pedicel abscission and fruit abscission/loss (Ozga et al., 2017). Developing seeds of pea and pericarp contains GAs and auxins (4-Cl-IAA and IAA) (Rodrigo et al., 1997; Ozga et al., 2017). Heat stress leads to seed abortion by altering seed-derived auxins and other seed signaling molecules transported to the pericarp, potentially having a negative effect on pericarp growth and facilitating pedicel abscission.

Elevated temperatures during seed filling and maturation can increase the proportion of seeds that are shriveled and abnormal at physiological maturity and result in seeds that exhibit reduced germination and seedling vigor in soybean (Egli et al., 2005). Furthermore, in legumes such as soybean, heat stress leads to the retention of chlorophyll in mature seeds, which can reduce seed quality (Teixeira et al., 2016). Low leaf photosynthetic rates during seed filling in heat-stressed plants are a major cause of reduced seed size (Awasthi et al., 2014). The accumulation of various seed components (mainly starch and proteins) may be inhibited by heat stress due to inactivation of enzymatic processes involving starch (Ahmadi and Baker, 2001) and protein synthesis (Triboï et al., 2003). Reduced seed weight was associated with reduced starch biosynthesis enzyme activities (ADP-glucose pyrophosphorylase and soluble starch synthase) in the endosperm during seed filling when the temperature increased above a threshold level (Singletary et al., 1994). Heat stress also reduces invertase activity associated with reduced carbon degradation (from sucrose to hexose) and partitioning (to starch synthesis) within endosperm, rather than being associated with limited carbon supply to the seed (Ozga et al., 2017). The legume embryo, being a strong terminal sink for sucrose, is not vascularly connected to the maternal seed coat tissue (Hardham, 1976). In faba bean, Weber et al. (1996), proposed a model for invertase-mediated unloading of sucrose for legume embryos during early seed development. Heat stress interrupts seed invertase activity and may alter nutrient portioning and seed growth and maturation in legumes (Ozga et al., 2017). During seed development and maturation, hormone regulation plays an important role in legume (Jameson and Song, 2016). Heat stress reduces cytokinin levels in seed leading to reduced seed cell numbers and growth rates (Emery et al., 2000; Jameson and Song, 2016). According to Yang et al. (2016), treatment with CK (6-benzylaminopurine) diminishes the inhibitory effect of heat stress on seed filling rate, division rate of endosperm, endosperm cell number, and seed weight in soybean. Heat stress regulates GA biosynthesis and catabolism in developing seeds to reduce GA-associated seed growth and development processes (Ozga et al., 2017). High-temperature stress increases the levels of ethylene, leading to reduced growth and enhanced senescence and abscission of various plant organs (Kukreja et al., 2005; Abeles et al., 2012). Heat stress induces ethylene, which can reduce photosynthesis and grain filling rates, and cause embryo abortion in some crops such as wheat (Rajala and Peltonen-Sainio, 2001; Hays et al., 2007). The effects of heat stress on different growth hormones at various reproductive developmental stages in legumes are listed in **Table 3**.

**Table 3:** Effects of heat stress on different growth hormones at various reproductive developmental stages in legumes.

Legumes	Growth hormone	Stage of development	Effects	Reference
Pea	Auxin	Stamen and pollen development	Represses auxin biosynthesis and signaling in developing anthers, resulting in pollen developmental abnormalities	Abeysingha, 2015
Common bean	Ethylene	Stamen and pollen development	Affects ethylene biosynthesis/signaling pathways in the developing anther, which leads to reduced anther dehiscence	Porch and Jahn, 2001
Soybean	Auxin and ethylene	Fruit set	Reduces auxin flux through the pedicel, allowing ethylene-facilitated pedicel abscission and fruit loss to occur	Oberholster et al., 1991
Pea	Auxins and gibberellins	Fruit set	Induces seed abortion, likely to affect the level of seed-derived auxins, and other seed signaling molecules transported to the pericarp, potentially having a negative effect on pericarp growth and facilitating pedicel abscission	Ozga et al., 2009
Lupins	Cytokinins	Seed development and maturation	Reduces seed CK levels leading to reduced seed cell numbers and seed growth rates	Emery et al., 2000
Pea	Gibberellins	Seed development and maturation	Modulates GA biosynthesis and catabolism in developing seeds in a similar manner to that observed in vegetative tissues; reduces GA-associated seed growth and development processes	Stavang et al., 2005
Chickpea	Ethylene	Seed development and maturation	Protects plants against heat stress-induced oxidative damage, possibly by acting as a signal to activate oxidative defenses	Kukreja et al., 2005


### Physiological and Metabolic Basis for Reproductive Failure under Heat Stress

There are limited studies on the response of stage-specific functional physiology from flowering and post-flowering in legumes during high-temperature stress. Though the susceptibility to heat stress in plants varies with plant development, the reproductive stage due to its sensitive organelle makeup is bound to experience greater impact and surrender to temperature vagaries. The response depends upon the species and genotype, with profuse inter- and intra-specific differences (Sakata and Higashitani, 2008; Bita, 2016). Heat stress alters photosynthesis and respiration to shorten the life cycle and thus to reduce the plant productivity (Barnabás et al., 2008). A reduction of photosynthesis will in due course deplete the energy reserves and limit the availability of resources for reproduction in parental and gametophytic tissues (Sumesh et al., 2008). Heat stress often hastens the onset of anthesis, to start the reproductive phase of development before ample resources are gathered (Zinn et al., 2010). Several genes are alterted under high-temperature stress to result in degenration of tapetum and pollen sterility in many plant species (Oshino et al., 2007; Endo et al., 2009). Elevated temperatures target the enzymes involved in carbohydrate metabolism (e.g., cell wall, vacuolar invertase, and sucrose synthase) and sugar-transporters to reduce the pollen viability (Hedhly, 2011). Particularly, enzymes invertase and sucrose synthase isomorphs are down-regulated, which affects the turnover of starch and sucrose in pollen grains to decrease accumulation of soluble carbohydrates (Hedhly, 2011).

Male sterility has been observed in many heat-stressed food legumes, such as chickpea (Kaushal et al., 2013) and mungbean (Kaur et al., 2015), and impaired pollen development has been a vital reason linked to yield losses due to heat stress (Wassmann et al., 2009). Anthers developing under high temperature showed cell-proliferation arrest, distended vacuoles, altered chloroplast development and mitochondrial abnormalities (Sakata et al., 2010). Heat stress decreases accumulation of carbohydrates in pollen grains and stigmatic tissue by changing partitioning of the assimilates and the proportion between symplastic and apoplastic loading of the phloem (Taiz and Zeiger, 2006), which affects pollen viability (Kaushal et al., 2013). Heat stress decreases the activity of sucrose synthase and many cell wall and vacuolar invertases in developing pollen grains; as a result, the turnover of sucrose and starch turnover is impaired to reduce the accumulation of soluble carbohydrates in chickpea (Sato et al., 2006; Kaushal et al., 2013). Similar findings have been reported in chickpea (Devasirvatham et al., 2012a; Kaushal et al., 2013), lentil (Bhandari et al., 2016), and mungbean (Kaur et al., 2015). Heat stress also decreases the starch, protein and total oil yield in many crop species such as soybean (Rotundo and Westgate, 2009; Thuzar et al., 2010), and has been linked to high temperatures during seed development (Banowetz et al., 1999). Thus, for crop production under elevated temperatures, it is highly desirable to know which developmental stages and plant processes are most sensitive to heat stress, as well as whether high day or high night temperatures are more detrimental.

## Physiological Responses

Heat stress may result in many physiological abberations such as leaf and stem scorching, leaf abscission and senescence, shoot and root growth inhibition, and fruit damage, which consequently lead to reduced plant productivity (Vollenweider and Günthardt-Goerg, 2005). The initial impacts of thermal stress involve structural alterations in chloroplast protein complexes and reduced enzyme activity (Ahmad et al., 2010). Heat stress at the cellular level leads to membrane damage, protein denaturation, enzyme inactivation in mitochondria and chloroplasts, impaired protein and carbohydrate synthesis, protein degradation, new protein synthesis, and impaired carbon metabolism (Schoffl et al., 1999; Kaushal et al., 2013). Further, heat stress alters the permeability of membranes by direct injuries, impacts the differentiation, elonagtion and expansion of cells by changing the organization of microtubules and eventually to the cytoskeleton (Rasheed, 2009; Bita and Gerats, 2013).

### Membrane Damage

Among the components of a plant cell, plasma membranes are considered the most heat-sensitive, as they are the primary sites of injury under heat stress (Blum, 1988; Wise et al., 2004). Elevated temperature severely affects membrane structure and function, thereby increasing the fluidity of membranes due to protein denaturation and increased unsaturated fatty acids, causing a phase transition from solid gel structure to flexible liquid crystalline structure (Wahid et al., 2007). Due to the presence of double bonds in fatty acids (unsaturated state), these are less tightly packed into a membrane (Horváth et al., 2012), which facilitates the activation of lipid-based signaling cascades, elevated Ca^2+^ influx and reorganization of cytoskeletal (Ruelland and Zachowski, 2010; Bita and Gerats, 2013). Heat stress injury can be determined by loss of membrane integrity due to structural modifications of component proteins, which increase membrane thermostability and leakage of organic and inorganic ions from cells (Salvucci and Crafts-Brandner, 2004). Therefore, an electrolyte leakage value serves as an indicator of membrane damage and reflects stress-induced changes and has been used to evaluate membrane thermostability under high-temperature stress conditions (Liu and Huang, 2000; Xu et al., 2006). The effects of heat stress on membranes have been reported in various legume crops. In soybean, enhanced membrane permeability and electrolyte leakage was noticed under heat stress (Lin et al., 1984), which decreased the capacity of the plasma membrane to hold water and solutes. Similarly, membrane injury was noticed in chickpea genotypes, especially sensitive genotypes, at 40/30°C, which was further intensified at 45/35°C (Kumar et al., 2013). Chickpea is the most heat sensitive legume, as per observations based upon membrane thermostability and PSII function, compared with other grain legumes such as pigeon pea, groundnut, and soybean (Devasirvatham et al., 2012b). Other cool-season legumes such as faba bean and lentil have also been evaluated for membrane thermostability, which is closely related to plant heat tolerance (Ibrahim, 2011; Barghi et al., 2012). Membrane thermostability has been successfully employed to assess thermotolerance in many food crops worldwide.

### Photosynthesis

Structural changes in thylakoid membranes with moderately high temperature stress have been observed using the freeze-fracture technique (Gounaris et al., 1984; Sharkey, 2005). The three major heat-sensitive sites in photosynthetic machinery are the photosystems, mainly photosystem II (PSII) with its oxygen-evolving complex (OEC), and the ATP generating and carbon assimilation processes (Mohanty et al., 2007; Murata et al., 2007). Photosystems I and II show damage under high temperature, with PSII more sensitive in chickpea (Kaushal et al., 2016). PSII in the electron trasnport chain of light reaction (Heckathorn et al., 2002) and RuBisCO activase in the carbon fixation cycle (Crafts-Brandner and Salvucci, 2000) are both sensitive to high temperature (Sinsawat et al., 2004; Kaushal et al., 2013). Heat stress damages the chlorophyll and photosynthetic apparatus by producing ROS (Guo et al., 2007; Bita and Gerats, 2013). In chickpea, Kumar et al. (2013) reported that damage to chloroplast membranes, mainly due to deterioration of photosynthetic pigments, reduced photosynthesis under high-temperature stress. A reduction in chlorophyll under elevated temperature has been reported in beans (Petkova et al., 2007) and chickpea (Kumar et al., 2013).

Higher temperature reduces the photosynthetic rate by decreasing leaf chlorophyll and nitrogen contents. In soybean, heat stress (38/28°C) significantly reduced chlorophyll content and, as a result, sucrose content. High-temperature stress reduces carbohydrate synthesis and carbohydrate transport from leaves; as a result, carbohydrates are diverted into vegetative organs at the expense of reproductive organs (Plaut et al., 2004; Suwa et al., 2010; Zhou et al., 2016). Heat stress negatively affects photosynthesis, carbohydrate synthesis, and flower and bud numbers, and ultimately leads to reduced sucrose content, the primary end product of photosynthesis translocated to reproductive organs (Lalonde et al., 1999). Leaf photosynthesis directly affects sucrose import into reproductive organs (Boyer and McLaughlin, 2007). Sucrose import and utilization are affected by invertase activity (breaks down sucrose), which regulates carbon allocation and sugar signaling (Roitsch and González, 2004), and could affect flower and fruit set due to high-temperature stress (Zhou et al., 2016), as observed in chikcpea (Kaushal et al., 2013). The effects of high temperature on the process of photosynthesis in some legume crops are listed in **Table 4**.

**Table 4:** Effects of high temperature on the process of photosynthesis in some legume crops.

Plant	Temperature	Effects	Reference
Soybean	42/43°C	Damaged PSII	Ferris et al., 1998; Li S.J. et al., 2009; Li W. et al., 2009
		Reduced Fv/Fm	
Soybean	45/40°C	Damaged PSII	Srinivasan et al., 1999
Broadbean	42°C	Decreased photosynthesis	Hamada, 2001
Beans	30°C	Reduced Q_10_	Pastenes and Horton, 1996
Beans	30–35°C	Limited carbon assimilation and reduced supply of NADPH	Pastenes and Horton, 1996
Sorghum	40/30°C for 45 days	Decreased photosynthetic rate	Djanaguiraman et al., 2010
Chickpea	45/35°C	Inhibited chlorophyll content and photochemical efficiency; reduced photosynthesis and Fv/Fm	Kumar et al., 2013
Chickpea	Above 32/20°C	Reduced RuBisCO and sucrose activities	Kaushal et al., 2013
Chickpea	45/35°C	Damaged PSII	Srinivasan et al., 1999
Soybean and bird’s foot trefoil	Above 40°C	Disrupted normal functioning of PSII and impaired structure and functioning of related proteins and enzymes	Sainz et al., 2010; Board and Kahlon, 2011
Soybean	38/28°C	Reduced Chl content (by 18%) and photosynthesis (to 20%)	Tan et al., 2011
Groundnut	45/40°C	Damaged PSII	Srinivasan et al., 1999
Faba bean	30–40°C	Decreased chlorophyll variable, reduced photosynthetic rate, impaired chloroplast activity	McDonald and Paulsen, 1997
Lentil	30–35°C	Limited electron flow	Redden et al., 2014
Mungbean		Impaired photosynthetic efficiency	Bansal et al., 2014
Mungbean	>40°C	Decreased sucrose in leaves due to reduced RuBisCO activity and sucrose synthesizing enzymes	Bindumadhava et al., 2017
Pea	25–35°C	Decreased photosynthetic activity	Haldimann and Feller, 2005
Pea	40°C	Inhibited electron donation by OEC	Oukarroum et al., 2013
Pigeon pea	45/40°C	Damaged PSII	Srinivasan et al., 1999


### Water Relations

Heat stress is frequently associated with rapid loss of water from the plant surface resulting in dehydration (Koini et al., 2009). Heat-induced hikes in transpiration and water movement is a necessary tool for plant survival under extreme temperatures (Kolb and Robberecht, 1996; Hasanuzzaman et al., 2013). Increased transpiration during the day siphons out excess moisture from plants resulting in reduced turgor pressure and ultimately disturbed key physiological processes (Tsukaguchi et al., 2003). High-temperature stress influences plant water relations due to the faster depletion of water from soil profiles which affects soil temperatures and transpiration. High-temperature stress indirectly affects osmotic adjustments through impaired photosynthesis (especially damage to PSII), increased respiration, reduced leaf osmotic potentials, and decreased sugar concentrations (Huve et al., 2005; Vara Prasad et al., 2008). In snap bean (*Phaseolus vulgaris*), under heat stress, loss of water during the day time was more common because of increase in trasnpiration than night time, resulting in generation of water deficit stress (Tsukaguchi et al., 2003). Leaf transpiration rate increases once the threshold temperature is reached increase leaf cooling under heat stress (Levitt, 1980). High stomatal conductance under heat stress enhances transpirational heat dissipation in tolerant chickpea genotypes as long as soil water is available (Kaushal et al., 2013). However hastening drought stress will have further physiological implications, not least on photosynthesis (Liu et al., 2004). On the other hand, under severe heat stress, stomatal conductance decreases markedly, as in tobacco (Tan et al., 2011) to agagravate the damage to leaves.

### Nitrogen Fixation

Drought and heat stress conditions in the semi-arid tropics restricted nitrogen fixation efficiency (Naya et al., 2007). Elevated temperatures can affect N_2_ fixation directly or indirectly. Direct inhibition by temperature is a consequence of decreased nodule development (Dart and Mercer, 1965; Piha and Munns, 1987; Junior et al., 2005), functionality (Hernandez-Armenta et al., 1989) and accelerated nodule senescence whereas indirect inhibition is related to temperature effects on root hair formation, reduction of nodulation sites (Frings, 1976), and modified adherence of bacteria to root hairs (Frings, 1976). Heat stress impacts on rhizobia have been thoroughly studied (Lira et al., 2005). The maximum temperature for rhizobial growth ranges from 32 to 47°C (Hungria and Vargas, 2000). Rahmani et al. (2009) established that heat tolerance in *Bradyrhizobium* directly affects the symbiotic efficiency between *Bradyrhizobium* and the soybean host. All stages of legume–rhizobium symbiosis are susceptible to high temperature (Hungria and Vargas, 2000; Nehra et al., 2007; Yadav and Nehra, 2013). Hungria and Franco (1993), studied the effect of high-temperature exposure on nodulation and efficiency of N_2_ fixation in common beans; under high-temperature treatment (35 and 38°C/8 h/day), nodules formed but were inefficient at N_2_ fixation. The control plants (grown at 28°C), when exposed to even higher temperatures (40°C/8 h/day) at flowering, had reduced nitrogenase activity and N_2_-fixation efficiency. No nodules formed in peanut at 40°C or soybean at 37°C (Hungria and Vargas, 2000). Therefore, the selection of temperature tolerant N_2_-fixing rhizobial strains may be used as an efficient tool for mitigating temperature stress (Yadav and Nehra, 2013).

## Phytohormones and Signaling Molecules

Various phytohormones (ABA, brassinosteroids, etc.) as well as many signaling molecules (nitric oxide, etc.) purportedly play important roles under heat stress to confer heat tolerance (Hasanuzzaman et al., 2013; Asthir, 2015). Interactive effects of ABA and osmolytes were investigated in chickpea; exogenous application of ABA (2.5 μM) considerably alleviated seedling growth at 40/35 and 45/40°C by enhancing the levels of osmolytes (Kumar et al., 2012b). ABA-treated *Phragmites communis* plants had less oxidative damage than their non-treated counterparts, and reduced levels of MDA and H_2_O_2_ and increased levels of SOD, CAT, APX, POX (Ding et al., 2010). High temperatures of 35/25 and 45/35°C (as day/night 12 h/12 h) applied to chickpea plants under controlled environment, resulted in increased activities of antioxidants, such as glutathione, and proline (Kumar et al., 2011). Exogenous application of 2.5 μM ABA at 35/30, 40/35, and 45/40°C as day/night increased growth, reduced oxidative damage and decreased MDA and H_2_O_2_ concentration in chickpea (Kumar et al., 2012b).

Brassinosteroids (BRs) improved the growth and biomass of French beans under heat stress (Upreti and Murti, 2004) by stimulating cell elongation (Salchert et al., 1998). Vegetative growth, total yield and quality of pods, and total phenolic acids in pods increased in *Phaseolus vulgaris* after spraying with 25 and 50 mg L^-1^ BRs at 34.7–35.2 and 25°C (El-Bassiony et al., 2012). Salicylic acid (SA) is a natural derivative of phenols formed by phenylpropanoid metabolism. It is an important signaling molecule under stress conditions and an effective protectant under heat stress (Yuan Z.C. et al., 2008; Hasanuzzaman et al., 2013). SA modifies the activity of many enzymes and enhances chlorophyll and carotenoid level along with photosynthetic rates. In addition, SA has improved plant growth, flower induction, ion uptake and thermogenesis, and can affect stomatal movement and ethylene biosynthesis (Hayat et al., 2009).

Plants pre-treated with SA showed enhanced heat tolerance in some species (Clarke et al., 2004; Larkindale and Huang, 2004). In heat-stressed mungbean seedlings, pre-treatment with SA decreased lipid peroxidation to improve membrane thermostability and antioxidant activity (Saleh et al., 2007). Pan et al. (2006) observed an increase in endogenous levels of SA in pea plants in response to heat stress. SA applied exogenoulsy at 0.1–0.5 mM checked wilting in common beans and tomato under heat stress (Senaratna et al., 2000).

Nitric oxide (NO) is an important concentration-dependent and redox-related signaling molecule (Fancy et al., 2017). NO regulates various physiological processes and has a vital role in conferring tolerance to plants under abiotic stress including heat stress (Hasanuzzaman et al., 2010, 2011, 2012, 2013; Waraich et al., 2012). Treatment of heat-stressed mungbean plants with NO as sodium nitropruside assisted in maintaining the stability of chlorophyll a fluorescence, membrane integrity, H_2_O_2_ content, and antioxidant enzyme activity (Yang et al., 2006).

## Genetic Approaches for Heat Tolerance in Legumes

The deleterious effects of abiotic stresses on agricultural productivity can be minimized through a combination of cultural practices and genetic improvement. Genetic improvement can develop cultivars that perform better under high temperatures leading to improved economic yields (Tilman et al., 2002; Varshney et al., 2011). In the field, screening for heat stress tolerance faces significant challenges due to interactions with other environmental factors, but multiple screenable traits are available for successful selection (Hall, 2011). Heat-tolerant genotypes can be selected under controlled conditions, which although expensive but do not allow other factors to interfere that interact with the high-temperature tolerance mechanisms under field conditions (Souza et al., 2012). The development of an effective set of thermotolerance markers is the key for breeders, which can be used further to confer tolerance (Bita and Gerats, 2013). The development of superior varieties with increased tolerance requires an understanding of the response mechanisms for stress in legumes, including variations in gene expression and the resultant changes in the transcriptome, metabolome, and proteome (Ramalingam et al., 2015). Due to the limited number of genetic inheritence studies, there exist less understanding of genetic basis of high temperature tolerance in grain legumes (Jha et al., 2017). Various genetic analysis have been performed based on the Mendalian and quantitative genetics to unravel the genetic basis of heat stress tolerance in legumes (Patel and Hall, 1988; Baiges et al., 1996; Miklas et al., 2000). At first, in grain legumes genetic inheritence of essential agronomic traits contributing to yield performance, directly or indirectly, under high temperature stress and governed mainly by major/single has been worked out (Patel and Hall, 1988; Hall, 1993). For example, in cowpea genetic control of heat tolerance was attributed to single gene on the basis of analysis of various traits such as number of pods per peduncle and proportion of tolerant plants under high temperature stress in contrasting populations derived from heat-sensitive (Barnbey 23, “Magnolia” and 7964) and heat-tolerant (“Prima” and TVu4552) genotypes (Marfo and Hall, 1992). Through analysis of various traits such as pods/plant, seeds/plant, and seed weight in heat-tolerant genotypes multiple mechanisms for thermotolerance were unvieled in common bean (Rainey and Griffiths, 2005). Thus, by deciphering the genetic basis of thermotolerance, performance of plants under stress conditions can be improved leading to their enhanced performance.

### Conventional Breeding Approach toward Heat Tolerance

Traditional breeding programs focus on developing cultivars with high yield traits under non-stress conditions. Such efforts have helped to enhance crop production per unit area and increased total agricultural production (Warren, 1998). Plant improvement for heat stress tolerance through genetic engineering is an economically better solution for crop production under stressful conditions (Blum, 1988). Heat sensitivity varies across developmental stages which makes the development of thermotolerant crops a challenging task (Driedonks et al., 2016). While breeding approaches have made significant advances in generating heat-tolerant lines in various crops, the genetic basis and range of heat tolerance largely remain unrevealed, especially in legumes. Development of new varieties is time-consuming and costly; therefore, understanding heat tolerance mechanisms would facilitate in developing strategies for screening germplasm of various legumes for traits related to heat tolerance. Using and exploring wild varieties along with landraces in breeding will enhance genetic diversity in crops (Driedonks et al., 2016).

High-temperature tolerance through conventional breeding is one approach to minimizing the damaging effects of heat stress on crop yield (Krishnamurthy et al., 2011). Breeding programs are generally conducted in a climactic region having similarity to where the crop will be grown. For relatively hot regions, selection of breeding lines occurs under hot conditions (Mickelbart et al., 2015). This technique has been reasonably successful considering that crops grown in warmer regions are often more tolerant of high temperatures than those in cooler regions (Kugblenu et al., 2013; Gaur et al., 2014). The chickpea genotype ICCV 92944, which is heat tolerant in screening experiments, has been released in three countries (as JG14 in India, Yezin6 in Myanmar and Chinadesi2 in Kenya) (Gaur et al., 2014). Two faba bean varieties (Shendi and Manami) with heat tolerance have been released in Sudan (Gaur et al., 2014). A new variety of cowpea has been produced with higher grain yield during high temperatures in the reproductive stage (Ehlers and Hall, 1998). Many heat-tolerant genotypes of legumes have been developed using various conventional breeding methods. By using rapid generation advancement methods, heat-tolerant index and earlier empirical methods, tolerant chickpea genotypes have been developed (Gaur et al., 2008; Krishnamurthy et al., 2011). Heat-tolerant common bean has been developed using the stress tolerant index (STI), geometric mean (GM) and recurrent selection techniques (Porch, 2006). Sultana et al. (2012) developed heat-tolerant genotypes of lentil using rapid initial growth habit and earliness. Mungbean, pea and snap bean have also been made tolerant to heat stress using the temperature-induction response and pedigree methods, respectively (Porch and Hall, 2013; Bindumadhava et al., 2017). Other crops such as groundnut and cowpea have been developed for improved performance under elevated temperatures using varied conventional breeding methods namely solute leakage, chlorophyll fluorescence and STI (in the case of groundnut), cross combination, breeding, pedigree breeding/backcrossing, and pedigree method (cowpea only) (Patel and Hall, 1990; Hall, 1992, 1993, 2011; Craufurd et al., 2003; Lucas et al., 2013).

While conventional breeding has been successful in developing heat-tolerant lines, the physiological and genetic basis of improvement remains unsure. This prevents the identificationof molecular biomarkers which would help in screening germplasm for enhanced heat tolerance and permit effectual breeding of this complex trait. Moreover, in conventional breeding, the potential gain in tolerance to heat stress is restrained by low genetic diversity (Paran and van der Knaap, 2007). Genetic diversity exists for heat tolerance in legumes (Kumar et al., 2016; Bindumadhava et al., 2017). Legume breeding programs, with various classical breeding methods, have potential in the application of technology that could promote their global production.

### Genetic and Quantitative Trait Locus (QTL) Mapping

Genetic and quantitative trait locus (QTL) mapping has become a successful method for detecting specific chromosome segments that have candidate genes for heat tolerance (Argyris et al., 2011; Zhang et al., 2012). To improve knowledge regarding heat tolerance at the genetic level, attempts have been made to identify QTLs in segregating mapping populations. Till now a wide range of QTLs governing heat tolerance has been discovered in cereal crops (Chen et al., 2008; Zhang et al., 2008; Kumar et al., 2010; Mason et al., 2010; Wei et al., 2013), but very few heat-tolerant QTLs have been reported so far in legumes mainly including cowpea (Lucas et al., 2013; Pottorff et al., 2014) and azuki bean (Kaga et al., 2003; Vaughan et al., 2005). QTLs for several traits related to heat tolerance have been identified, such as increased chlorophyll fluorescence and reduced canopy temperature during vegetative and reproductive stages in wheat (Vijayalakshmi et al., 2010; Lopes et al., 2012). Reduced canopy temperatures show that efficient water uptake is ultimately associated with deep rooting, and increased chlorophyll fluorescence reflects heat-tolerant photosynthesis (Pinto and Reynolds, 2015). Studies have been conducted on the effect of heat stress on reproductive characters, mainly pollen germination, pollen tube growth, grain filling, grain weight, fruit set and post-anthesis senescence of leaves (Driedonks et al., 2016). A QTL study on rice (*Oryza sativa*) recently focused on spike fertility under heat stress (Ye et al., 2015). This study was based on earlier work (Ye et al., 2012) and confirmed that a recessive QTL on chromosome 4 is present, which is responsible for a 15% increase in rice spikelet fertility under high temperatures (Ye et al., 2015). The use of a multiparent advanced generation inter-cross (MAGIC) population may lead to the introduction of more genetic variation and identification of thermotolerant genes for spikelet fertility (Ye et al., 2015).

Quantitative trait locus can also be dedicated to the investigation of natural populations. As observed earlier, linkage mapping may be able to detect crucial genes and QTLs. However, the restricted number of generations and recombination events often results in QTLs covering a comparatively large region and the identification of genes involves a tedious process of fine mapping (Driedonks et al., 2016). Therefore, fine mapping is generally inefficient for the detection of candidate genes (Bergelson and Roux, 2010). Different studies on QTLs revealed multiple QTLs per trait, ranging from two in azuki bean and rice (improved pollen viability and spikelet number under high temperatures, respectively) to 34 in barley for traits related to heat stress. As such, heat tolerance depends on a variety of factors and QTLs, which differ among the crops (reviewed in Jha et al., 2014). Kaga et al. (2008) identified HQTL-1 and HQTL-2 in azuki beans involving traits for pollen viability. In cowpea, many QTLs have been detected, in particular Hbs-1, Hbs-2, and Hbs-3 for heat-induced browning of the seed coat (Pottorff et al., 2014), afot 1.1, afot 1.2, afot 1.3 and afot 2 for flower opening (Andargie et al., 2013), and Cht-1, Cht-2, Cht-3, Cht-4, and Cht-5 for male heat sterility (Lucas et al., 2013). In pigeon pea, qPD4.1 have been detected for pods per plant, and qFL4.1 and qFL5.1 for flowering (Kumawat et al., 2012). Currently, association mapping is acquiring popularity as a trait mapping technique which complements conventional QTL mapping (Yu et al., 2008; Jha et al., 2017). Recently, GWAS (genome-wide association study) was carried out in a panel of 300 accessions to scrutinize the marker-trait association for thermotolerance in chickpea (Thudi et al., 2017). Therefore, to accelerate the transfer of heat tolerance causative gene(s)/QTL(s) in major grain legumes, available molecular markers can be used in marker-assisted breeding programs (Jha et al., 2017). Futher, unrivaled improvements in next-generation sequencing (NGS) has paved way to unfold the complex genomic regions which are important in regulating complex traits (Elshire et al., 2011; Edwards and Snowdon, 2013). Genotype-by-sequencing (GBS) is one such technology that produces large number of SNP markers (Elshire et al., 2011), which are applied to develop genetic maps and decipher complex traits in legumes (Jaganathan et al., 2015; Kujur et al., 2015; Tayeh et al., 2015; Verma et al., 2015). The rising availability of refrence genome sequences in many grain legumes such as mungbean (Kang et al., 2014), soybean (Schmutz et al., 2009), groundnut (Bertioli et al., 2016; Chen et al., 2016), chickpea (Jain et al., 2013; Varshney et al., 2013), adzuki bean (Kang et al., 2015; Yang et al., 2015), pigeonpea (Varshney et al., 2012a), and common bean (Teixeira et al., 2005; Schmutz et al., 2014), provide great endevours to focus on important agricultural traits including thermotolerance.

Quantitative trait locus analysis in heat-sensitive and tolerant crops is gaining attention. The main advantage of QTL-based approaches is that they allow loci linked to heat stress to be identified (Bita and Gerats, 2013). Identification of adaptive QTLs for heat stress is one way of understanding tolerance mechanisms, and various studies have been conducted to detect genetic markers for various abiotic stresses, including heat stress (Roy et al., 2011). Markers linked to QTLs could be used to enhance thermotolerance in available germplasm. Currently, QTL identification for thermotolerance is being carried out using different traits, such as thousand grain weight (TGW), canopy temperature depression (CTD), grain filling duration (GFD), yield (Pinto et al., 2010), and traits related to senescence (Vijayalakshmi et al., 2010).

Association genetics has recently been used to assist in QTL detection in various crop species (Ahuja et al., 2010). The markers associated with QTLs, once isolated, the candidate QTLs can be introgressed in elite lines via MAS technology. The traits are usually controlled by small effect QTLs or multiple pleiotropic genes which are the main drawback of generating tolerant genotypes for heat stress (Bita and Gerats, 2013). Marker-assisted recurrent selection (MARS), pyramiding various QTLs from a large number of populations in the same genetic background or GS (Genomic Selection) techniques can be used to overcome this (Tester and Langridge, 2010; Varshney et al., 2012b). The MAS approach, however, for complex traits such as heat tolerance are not efficient due to the genotype–environment and gene–gene interactions, which eventually lead to reduced breeding efficiency (Collins et al., 2008). When characters like heat stress tolerance are involved, recurrent selection is an adequate method in plant breeding. There is a small probability of obtaining a superior genotype in multiple crosses, which combines all of the required alleles. The substitute is recurrent selection to accumulate gradually, through recombination cycles, the desired and available alleles in different parents (Donà et al., 2013). The main focus of recurrent selection is to enhance the frequency of desirable alleles for favorable traits, conserving genetic variability.

### ‘Omics’ Technology in Heat Tolerance

‘Omics’ technologies, such as genomics, proteomics, transcriptomics, and metabolomics, have revolutionized research in plant sciences (Yuan J.S. et al., 2008). The enormous progress in the field of “omics” has made possible to elucidate different candidate genes involved in response to complex abiotic stresses in crop plants (Vij and Tyagi, 2007; Urano et al., 2010; Deshmukh et al., 2014; Kujur et al., 2015). These technologies involve various disciplines, and new advances in these areas have markedly contributed to a better understanding of the molecular and genetic basis of the heat stress response that has been a crucial bottleneck for molecular and transgenic breeding (Reddy et al., 2012). As the technology has progressed, omics approaches have improved over the last decade (Deshmukh et al., 2014). Research in recent years has provided an understanding of the function of proteins, metabolites, and many key genes and molecular processes involved in plant responses to heat stress. The mechanism of heat stress tolerance, however, is quite complex because of the multiple genes and post-transcriptional regulation influence (Ramalingam et al., 2015). Moreover, gene expression is affected by stress conditions due to alterations in plant proteome and metabolome composition. Therefore, to understand plant stress tolerance, omics technology has become mandatory (Ramalingam et al., 2015).

#### Transcriptomics

Various moden techniques such as RNA sequencing have led to many deep expression studies ultimately unraveling many heat-tolerant candidate genes in various crops (Xin et al., 2010; Wang et al., 2011; Priest et al., 2014; Gonzalez-Schain et al., 2016). Few studies have been conducted for heat tolerance via transcriptomic analysis in legumes. Initially cDNA – AFLP technique was used to analyze the expression of various thermotolerant genes in cowpea (Simões-Araújo et al., 2002). Owing to the importance of heat shock factors (HSF) for survival under heat stress, 19 and 21 HSF ESTs in *Lotus japonicas* and *Medicago truncatula* respectively and 25 candidate HSF ESTs in soybean were found (Soares-Cavalcanti et al., 2012). Kumar et al. (2015a) suggested that the transcript expression of VfHsp17.9CII gene in faba bean showed a considerable 620-fold change when subjected to high temperature treatment. Taking the advantage of NGS technology (which has made it possible to achieve greater resolution and improved description of candidate genes in trancriptome sequences) in ICC4958 genotype of chickpea DNAJ, HSP 70 and HSP 91 genes have been identified using Illumina/Solexa sequencing (Hiremath et al., 2011; Martin et al., 2013). In a recent experiment, employing RNA-sequencing, a complete trancriptome analysis of heat-responsive genes in heat-sensitive chickpea genotypes (ICC 5912, ICC 4567, and ICC 10685) and heat-tolerant genotypes (ICC 15614, ICC 1356, and ICC 92944) was reported (Kudapa et al., 2014). Later, in chickpea through RNA-sequencing analysis of leaf, flower and roots at different growth stages, five HSP 90 candidate genes (Ca_25811, Ca_23016, Ca_09743, Ca_17680, and Ca_25602) were obtained (Agarwal et al., 2016). To further explain the role of HSP 20 in thermotolerance, 47 genes of 51 GmHsp20 were identified based on an *in vivo* analysis to be heat responsive in soybean (Lopes-Caitar et al., 2013). Lee et al. (1994) cloned ClpB/HSP100 gene of soybean and unraveled evident underlying candidate gene Glma05 g00540. Later on, in soybean GmHsfA1 gene was cloned successfully which was responsible for thermotolerance (Chen et al., 2006; Zhu B. et al., 2006). VfHsp17.9-CII gene in faba bean (mainly belonging to sHSP CII) has been recently cloned (Kumar et al., 2015a). Increased accumulation of VfHsp17.9-CII at 38°C in pollen grains of faba bean was observed in this study thereby pointing out its protective role against heat stress in faba bean. It might be worthwhile to explore specific strategies to reduce ovary abortion as seen in maize with respect to drought stress induced seed loss (Guan and Koch, 2015). For example in case of maize, it has been found that increase in the expression of trehalose-6-phosphate phosphatase, the yield is improved under drought stress condition (Nuccio et al., 2015). Similar strategies should be looked in to the legumes growing under heat stress. Advancing trends in transcriptomics along with increasing knowledge about the sequence technologies coupled with improvements in computational tools would help us in understanding heat stress response in legumes.

#### Proteomics and Metabolomics

Proteomics and metabolomics are rapidly emerging fields that provide large-scale and precise information about the proteins and metabolites produced in response to various abiotic stresses in plants including legumes (Arbona et al., 2013; Rodziewicz et al., 2014; Ramalingam et al., 2015). In some model legume species such as *Medicago truncatula* and *Lotus japonicus*, along with crop legumes like soybean, proteome and metabolome profiling using high-throughput based systems have been used extensively to study nodule symbiosis, cellular and developmental processes, and stress signaling pathways. Furthermore, the available protein reference maps, proteomics, and metabolomics databases have been used extensively in research to unfold the various processes in legumes (Ramalingam et al., 2015).

During high temperature stress, protoemics study allow deciphering the role of heat-responsive proteins like HSPs or chaperones, proteins involved in various signal transduction pathways, redox homeostasis, metabolic pathways and protection (Kosová et al., 2011; Zou et al., 2011). The integration of proteomics with genetic information in legumes will give way to exciting opportunities to achieve crop improvement and sustainable agriculture (Rathi et al., 2016). The foremost challenge faced by proteomics is the presence of multiple proteins, all of which undergo various post-translational modifications (PTMs). Nonetheless, proteomics is proceeding quickly with a primary focus on PTMs, protein quantity and protein interactions (Champagne and Boutry, 2013).

Proteomics has made a major contribution to plant biological research and stress responses, mainly because of the increasing number of plant genomes being sequenced and released (Weckwerth, 2011; Jorrín-Novo et al., 2015). Additionally, mass spectroscopy advancements, bioinformatics, and quantitative methods have made it possible to comprehensively identify, validate and characterize a variety of proteins from specific organ/tissue/cells (Glinski and Weckwerth, 2006). The knowledge obtained from these advanced techniques is essential for interpreting the structure of proteins and regulatory functions of proteins encoded by specific genes (Wienkoop et al., 2010; Nanjo et al., 2011; Abdallah et al., 2012). Moreover, approaches like proteomics provide crucial information, such as the levels of proteins linked to stress tolerance, alterations in proteomes under stress conditions that associate analyses of transcriptomics and metabolomics, along with the role of genes expressed in the genome’s functionally translated regions linked to desirable traits (Kosová et al., 2011).

In legumes, proteomic studies have been mainly conducted on *Medicago* to understand stress tolerance, plant growth, and the physiology of seeds and development, which is of great importance to agricultural research (Colditz and Braun, 2010; Jorrín-Novo et al., 2015). There has been a considerable contribution to proteomic studies in soybean at subcellular, organ and whole plant levels, with 2D-GE (gel electrophoresis), MALDI-TOF-MS, LC–MS/MS and protein sequencing used to unravel the heat tolerance mechanism in soybean seedlings. Using these techniques in “heat-sensitive” soybean genotype, 42 protein spots were identified at different time scales that were involved in 13 metabolic processes (Wang L. et al., 2012). Further, proteomic analysis on leaves of soybean revealed the expression of 25 different proteins which have roles in important metabolic pathways, such as RuBisCo regulation, Calvin cycle, electron transport under high temperature (Das et al., 2016). In an experiment to highlight root proteome dynamics during heat stress, using normal root hairs and heat stressed root hairs, 30 commonly upregulated and downregulated proteins were obtained (Valdes-Lopez et al., 2016). Many peroxidases along with heat shock class I and II proteins were found in heat-treated soybean roots, indicating their role in heat tolerance. This information will allow further experiments to be conducted for proficient proteomics application for crop legumes, primarily by characterizing proteins linked with development and stress tolerance, to identify unambiguous candidate genes (Ramalingam et al., 2015). Similar reference maps have been obtained in crop legumes such as peanut and soybean. Some proteins (5702) have been identified for single root hair cells via proteome reference maps, generated in soybean (Brechenmacher et al., 2012). Development of proteome maps for chickpea, pigeonpea and groundnut is underway at ICRISAT.

Heuss-LaRosa et al. (1987) proposed role of two proteins (70 and 80 KD) in thermotolerance and adaptation in cowpea. In Mungbean, two HSP 70 isotypes were identified under heat stress (Wu et al., 1993). Zhu J. et al. (2006) observed the expression of HSP-interacting proteins for improved heat stress tolerance in soybean. In another study on, soybean seedlings, increased accumulation of various other proteins with chaperone functions (Chaperonin 60b subunit CPN60-b, HSP 90, Chaperonin CPN-10 and chloroplast chaperonin) occurred under heat stress (Ahsan et al., 2010). On the basis of differential expression of 35, 54, and 61 proteins from stems, leaves, and roots, respectively in response to high temperature role of tissue-specific proteins in safegaurding soybean against heat stress was reported (Ahsan et al., 2010). Role of ERD-related proteins (also serves as chaperones), HSP70 and HSP 91 in dehydration (and probably in thermotolerance) was observed in chickpea via trancriptome analysis (Hiremath et al., 2011). Presence of ClpB/HSP100 protein was detected under heat stress in *Phaseolus lunatus* (Keeler et al., 2000). It has been observed that accumulation of ClpB/HSP100 during high temperatures increased the pollen viability in faba bean (Kumar et al., 2015b). Recently, in faba bean VfHsp17.9-CII (a novel HSP protein) was identified which implements heat tolerance (Kumar et al., 2015a). Das et al. (2016) reported increased levels of Ef-Tu protein in soybean which are mainly involved in protecting key enzymes and proteins from heat stress that are required for photosynthesis. Therefore, the proteomic analysis of plants can unravel various underlying thermotolerant proteins that can further act as biomarkers in breeding program for producing thermotolerant grain legume varieties (Rathi et al., 2016).

Metabolomics, in addition to proteomics, is a vital approach to functional genomics that provides a method to identify and quantify metabolomes within a cell, tissue or organism (Weckwerth, 2003; Weckwerth and Kahl, 2013). Metabolomics plays a vital role in crop breeding programs as metabolites can be used as selection biomarkers because they can integrate complex interactions between genotype and environment (Fernie and Schauer, 2009). Tremendous progress in the field of metabolomics has made possible to achieve greater insights regarding various tolerance mechanisms at metabolic levels under heat stress (Kaplan et al., 2004; Obata and Fernie, 2012; Bokszczanin and Fragkostefanakis, 2013). Metabolite profiling performed in soybean genotypes revealed that anti-oxidants such as flavanoids, tocopherols, phenylpropanoids, and ascorbate refine heat tolerance in tolerant genotypes (Chebrolu et al., 2016). Little information exists on metabolomics for heat stress in plants, particularly legumes. This area needs to be exploited to comprehend the underlying mechanisms of heat stress (Ramalingam et al., 2015).

With proteomics and metabolomics emerging as powerful tools for unfolding various unknown plant mechanisms, there is great interest in applying these techniques to understand stress-related responses in crops (Pandey et al., 2016).

These advanced approaches along with genomics knowledge will support our efforts to accurately detect candidate genes and pathways responsible for important traits that will be invaluable for crop breeding programs (Langridge and Fleury, 2011). The information obtained from ‘omics’ studies will need to be combined with breeding so that breeders can move toward ‘knowledge-driven breeding’ as opposed to ‘chance-driven breeding’ (Kulwal et al., 2011). It is evident that these technologies will contribute to legume improvement programs in the future.

## Conclusion

Heat stress causes severe agricultural losses, which is a risk to world food security with consequences that will challenge human welfare. Among the crop growth cycle, the reproductive phase is more susceptible to high-temperature stress than the vegetative phase. While the male reproductive organs are more sensitive to heat stress than the female counterpart, the complete reproductive process from gamete formation to fertilization and seed maturation are highly vulnerable to raised temperatures. Microsporogenesis is disrupted at high temperatures due to damage caused by the tapetal layer and nutrient imbalance in developing pollen, resulting in sterility. Heat stress has detrimental effects on ovule development and viability. Fertilization is impaired due to reduced pollen viability, stigma receptivity, and pollen tube growth. Further, reduced seed filling, increased seed abortion and smaller seeds affect the seed weight. All these effects may occur due to diminished photosynthetic rates, which result from metabolic and cellular dysfunction, and lead to reduced photosynthate supply to developing seeds. During heat stress, plants undergo numerous adaptations which confer tolerance, such as the induction of signal cascade leading to profound changes in specific gene expression. Of the signaling molecules synthesized under stress conditions, Ca^2+^ plays a critical role. Heat shock proteins that accumulate and act as molecular chaperones help to fold and unfold proteins under heat stress. The application of ‘omics’ (genomics, transcriptomics, proteomics, and metabolomics) is essential for exploiting the molecular basis and processes underlying the plant response to heat stress and mechanisms of tolerance. Molecular-linked functional physiology will pave the way for engineering plants with improved tolerance, coupled with higher economic yields, to counter the harsh climates of arid to semi-arid zones of the world.

## Author Contributions

HN conceived the concept and BH supported the idea. AS and KS collected all the literature and compiled the information. AS, KS, and HN wrote the article. KHMS and BH extensively edited the article. RN, PV, SK, PG, MF, and RV, gave their valuable inputs in various specialized sections.

## Conflict of Interest Statement

The authors declare that the research was conducted in the absence of any commercial or financial relationships that could be construed as a potential conflict of interest.
